# Multidrug Aerosol Delivery During Mechanical Ventilation

**DOI:** 10.1089/jamp.2022.0057

**Published:** 2023-08-14

**Authors:** Ann D. Cuccia, Michael McPeck, Janice A. Lee, Gerald C. Smaldone

**Affiliations:** ^1^ Respiratory Care Program, School of Health Professions, Stony Brook University, Stony Brook, New York, USA.; ^2^ Pulmonary Mechanics and Aerosol Research Laboratory, Division of Pulmonary, Critical Care and Sleep Medicine, Department of Medicine, Stony Brook University Medical Center, Stony Brook, New York, USA.

**Keywords:** administration, aerosols, drug delivery, inhalation, mechanical, nebulizers and vaporizers, ventilators

## Abstract

**Background:**

In the critically ill, pulmonary vasodilators are often provided off label to intubated patients using continuous nebulization. If additional aerosol therapies such as bronchodilators or antibiotics are needed, vasodilator therapy may be interrupted. This study assesses aerosol systems designed for simultaneous delivery of two aerosols using continuous nebulization and bolus injection without interruption or circuit disconnection.

**Methods:**

One i*-AIRE* dual-port breath-enhanced jet nebulizer (BEJN) or two Aerogen^®^ Solo vibrating mesh nebulizers (VMNs) were installed on the dry side of the humidifier. VMN were stacked; one for infusion and the second for bolus drug delivery. The BEJN was powered by air at 3.5 L/min, 50 psig. Radiolabeled saline was infused at 5 and 10 mL/h with radiolabeled 3 and 6 mL bolus injections at 30 and 120 minutes, respectively. Two adult breathing patterns (duty cycle 0.13 and 0.34) were tested with an infusion time of 4 hours. Inhaled mass (IM) expressed as % of initial syringe activity (IM%/min) was monitored in real time with a ratemeter. All delivered radioaerosol was collected on a filter at the airway opening. Transients in aerosol delivery were measured by calibrated ratemeter.

**Results:**

IM%/h during continuous infusion was linear and predictable, mean ± standard deviation (SD): 2.12 ± 1.45%/h, 2.47 ± 0.863%/h for BEJN and VMN, respectively. BEJN functioned without incident. VMN continuous aerosol delivery stopped spontaneously in 3 of 8 runs (38%); bolus delivery stopped spontaneously in 3 of 16 runs (19%). Tapping restarted VMN function during continuous and bolus delivery runs. Bolus delivery IM% (mean ± SD): 20.90% ± 7.01%, 30.40% ± 11.10% for BEJN and VMN, respectively.

**Conclusion:**

Simultaneous continuous and bolus nebulization without circuit disconnection is possible for both jet and mesh technology. Monitoring of VMN devices may be necessary in case of spontaneous interruption of nebulization.

## Introduction

Continuous nebulization of selective pulmonary vasodilator aerosols during mechanical ventilation has become a common off-label therapy used in clinical practice for the management of refractory hypoxemia associated with acute respiratory distress syndrome (ARDS), severe pulmonary hypertension and, more recently, with COVID-19.^1–5^ Patients with severe asthma are also treated with albuterol infusion.^6^ These patients may benefit from additional aerosolized drugs such as added bronchodilators or antibiotics. In recent studies, our group has developed and assessed techniques for measurement of instantaneous aerosol delivery in a mechanical ventilation circuit to assess the effects of changing variables on drug delivery during continuous infusion and bolus delivery.^7,8^ The kinetics of routine bolus delivery of aerosols as well as more complex continuous infusion have been tested with different devices over a range of ventilator settings.

During continuous nebulization, coadministration of bolus therapy may require opening the ventilator circuit to add devices or disrupt continuous infusion resulting in clinical deterioration. Two nebulizer configurations have been proposed to avoid interruption of continuous nebulization: two stacked vibrating mesh nebulizers (VMNs) delivering two drugs, which have been previously reported in a case report^1^ and a single breath-enhanced jet nebulizer (BEJN) prototype outfitted with a dual-medication port adaptor that is currently in development and awaiting FDA 510(k) approval. This study evaluates these two technologies during simultaneous delivery of two aerosols during continuous nebulization and bolus injection. The experimental setup tests configurations that do not result in interruption of nebulization or circuit disconnection over a clinically relevant period (4 hours).

Real-time measurements with minute-by-minute determination of aerosol drug delivery will test the kinetics of a drug bolus injected during continuous nebulization to determine potential effects on the nebulizer and reliability of continuous therapy. The purpose of this study was to test the function of delivery systems designed to deliver multiple drugs; the “index case” the stacked Aerogen^®^ Solo VMN, which had been reported by Elnadoury et al.,^1^ but not tested, and a device designed specifically to provide both modalities in a simple single device (i-*AIRE* BEJN outfitted with a dual-medication port adaptor). To our knowledge, this delivery method has not been previously tested. We hypothesized that either a single i-*AIRE* BEJN with dual-medication port adaptor or two stacked Aerogen Solo VMN can be used to deliver multiple aerosols simultaneously without interruption or circuit disconnection and that concurrent bolus delivery does not change the overall qualitative and quantitative behavior of the continuous infusion.

## Methods

**FIG. 1.**
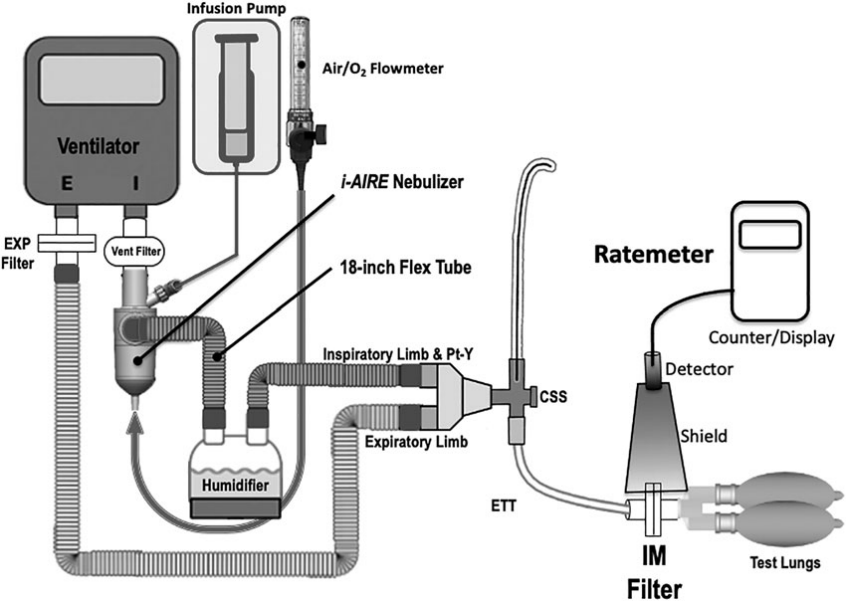
Experimental setup: The ventilator circuit during continuous infusion with the i*-AIRE* BEJN prototype with dual-medication port adaptor. BEJN, breath-enhanced jet nebulizer.

**FIG. 2.**
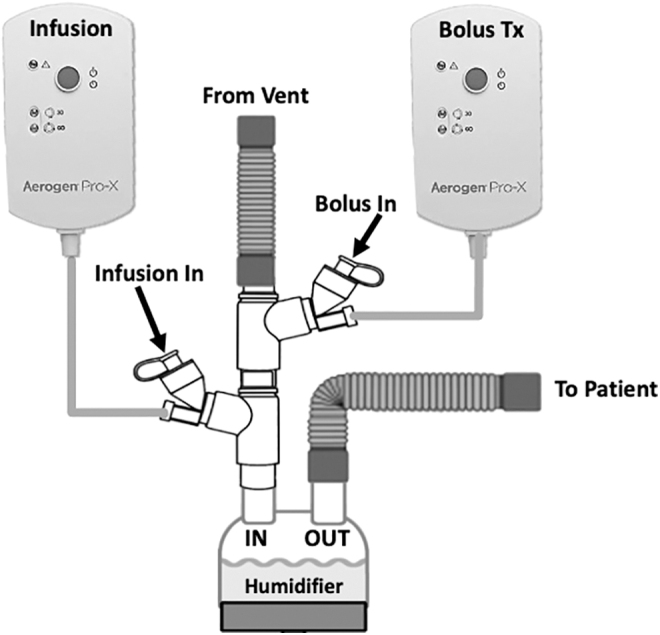
Stacked Aerogen Solo VMN and two controllers. One VMN and controller designated for continuous infusion (left) and designated bolus VMN and controller (right). VMN, vibrating mesh nebulizer.

The experimental setup is diagrammed in Figures 1 and 2. An Avea critical care ventilator (Vyaire Medical, Mettawa, IL) was used with two adult breathing patterns and two pump flows (5, 10 mL/h) (Table 1). The breathing patterns were selected to provide a clinically relevant range of duty cycles (DCs, inspiratory time %, determined as Inspiratory Time divided by Total Cycle Time). All tests were performed using the volume-controlled continuous mandatory ventilation mode (VC-CMV), with bias flow maintained at 2 L/min (DC 0.13: rate 15, tidal volume [VT] 450 mL, Ti 0.5 seconds, positive end expiratory pressure [PEEP] +5; DC 0.34: Rate 20, VT 650 mL, Ti 1.0 seconds, PEEP +5). Humidification was provided by a heated humidifier (MR-850; Fisher & Paykel Healthcare, Ltd., Panmure, Auckland, New Zealand) and dual-limb heated-wire ventilator circuit (Evaqua; Fisher & Paykel Healthcare, Ltd.) set at 37°C ± 1°C for all experiments.

**Table 1.** Experimental Conditions: Two Nebulizers, Two Breathing Patterns, Two Infusion Pump Flows, and Two Fill Volumes

Infusion pump flow (mL/h)	i-AIRE BEJN		Aerogen VMN	
	Breathing pattern Duty cycle (TI/TCT)		Breathing pattern Duty cycle (TI/TCT)	
	DC 0.13	DC 0.34	DC 0.13	DC 0.34
5	3 mL bolus 6 mL bolus	3 mL bolus 6 mL bolus	3 mL bolus 6 mL bolus	3 mL bolus 6 mL bolus
10	3 mL bolus 6 mL bolus	3 mL bolus 6 mL bolus	3 mL bolus 6 mL bolus	3 mL bolus 6 mL bolus
	DC 0.13: VC-CMV rate 15, VT 450 mL, Ti 0.5 seconds, PEEP +5 DC 0.34: VC-CMV rate 20, VT 650 mL, Ti 1.0 seconds, PEEP +5			

BEJN, breath-enhanced jet nebulizer; DC, duty cycle; PEEP, positive end expiratory pressure; TCT, total cycle time (TI+TE); TI, inspiratory time; VC-CMV, volume-controlled continuous mandatory ventilation mode; VMN, vibrating mesh nebulizer; VT, tidal volume.

The humidifier was operated in its invasive mode with default settings and the temperature display was allowed to reach 37°C before each experiment commenced. To complete the circuit, a closed suction system (Halyard Health, Alpharetta, GA) and a 7.5-mm endotracheal tube (Rusch; Teleflex Medical, Morrisville, NC) were connected to ventilate a pair of 1-L neoprene test lungs (Compliance 25.5 mL/cmH_2_O, Resistance: 20.4 cmH_2_O/L/s). An inhaled mass (IM) filter (Pari, Starnberg, Germany) placed in the circuit distal to the endotracheal tube measured the aerosol particles that would be inhaled by a patient (IM) under similar conditions.

Real-time measurement of aerosol delivery during mechanical ventilation was performed using a technique developed in our laboratory to assess the effects of changing variables on drug delivery during continuous nebulization.^9^ In brief, that technique involves a shielded ratemeter (Model 2200 Scaler Ratemeter; Ludlum Measurements, Sweetwater, TX) positioned at the distal tip of the endotracheal tube at the level of the IM filter for measurements of radiolabeled aerosol accumulating on the IM filter per minute over 4 hours. Ratemeter counts per minute were calibrated against quantitative units, for example, microcuries (μCi) of radioactivity with a dose calibrator or gamma camera.

After a room background radiation measurement coincident with the start of nebulization was obtained, the investigator triggered 1-minute counts of radioactivity every 2 minutes on the IM filter for the first 10 minutes and then every 5 minutes thereafter for each 4-hour experiment. For each 4-hour experiment, there were ∼42 experimental observations per experiment with each of the three output slopes reflecting ∼7–15 data points that were used for analysis and to establish each relationship.

The IM filter was changed at 90, 160, and 240 minutes, respectively, to avoid saturation. At the conclusion of the test run, a final 1-minute ratemeter count of the IM filter was made, followed by a measurement by gamma camera or dose calibrator to calibrate the ratemeter and to determine IM% for each data point.

Three InspiRx i-*AIRE* BEJNs molded with a dual-medication port adaptor (InspiRx, Somerset, NJ) and five Aerogen Solo VMNs (Aerogen, Galway, Ireland) were used in rotation for experiments. The experimental setup in Figure 1 shows the i-*AIRE* located at the ventilator outlet on the dry side of the humidifier. For Aerogen Solo experiments, the stacked Aerogen VMN system, first described by Elnadoury et al.,^1^ is shown with two devices and two controllers, one nebulizer and controller designated for continuous infusion and the other nebulizer and controller designated for bolus delivery (Fig. 2). A total of eight experiments were conducted for each nebulizer technology, one experiment for each condition (two experiments per each of the two breathing patterns at each pump flow) (Table 1).

Technetium-99m pertechnetate (^99m^Tc)-radiolabeled normal saline was utilized for all experiments, previous studies have demonstrated that normal saline is a suitable surrogate for multiple water-soluble drugs^10–14^ and that common drugs such as bronchodilators and steroids can be safely mixed.^15^ For continuous infusion experiments, a solution containing 9 to 16 mCi of ^99m^Tc was drawn into a 60 mL syringe to achieve ^99m^Tc concentrations of 150 to 270 μCi/mL. For the bolus treatment experiments, 3 and 6 mL solutions containing between 470 and 1330 μCi were prepared for injection. Before the start of each experiment, the nebulizer was dry, empty, and free of radioactivity.

Before each experiment, the radioactivity of the prepared solutions described above was measured with a radioisotope calibrator (Atom Lab 100; Biodex, Inc., Shirley, NY) to establish the initial charge. The total volumes of the prepared solutions were precisely measured to determine the initial radioactivity per unit volume. The time at which this initial charge was measured served as the baseline time for decay correction of the subsequent measurements obtained throughout the experiment.

**FIG. 3.**
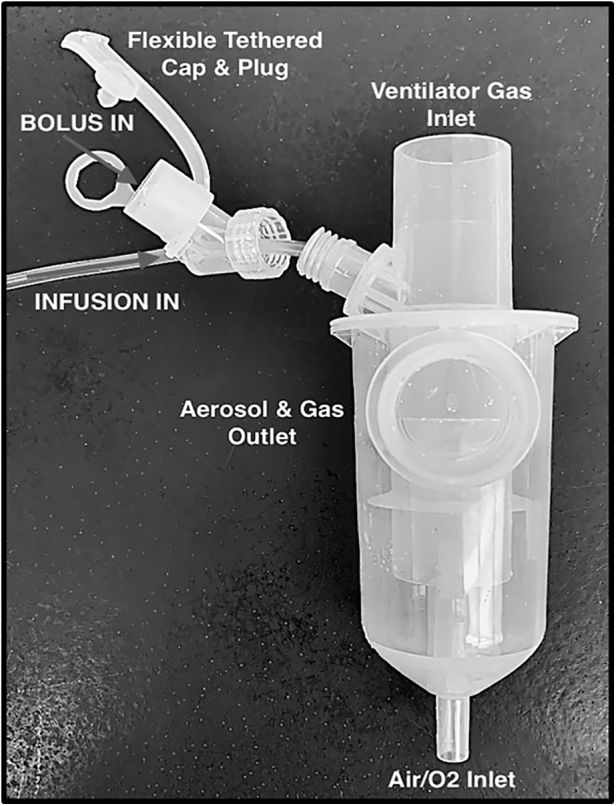
*i-AIRE* BEJN prototype with dual-medication port adaptor. Continuous infusion line is screwed on to nebulizer port with leur lock cap outfitted with medication port equipped with tethered cap and plug. Both nebulizer ports empty into a single nebulizer bowl.

First, the infusion pump was started at either 5 or 10 mL/h. At the same time, the Solo or i-*AIRE* devices were energized: the i-*AIRE* BEJN driven by compressed air at 3.5 L/min, 50 pounds per square inch gauge (PSIG, a measurement of tank gauge pressure with respect to atmospheric pressure), and Aerogen Solo VMN by each designated Aerogen controller. Ratemeter data were continuously monitored and recorded every 3–5 minutes (counts per minute [CPM]). After establishing continuous nebulization (over 30 minutes), the first bolus injection was made (3 mL) either into the bolus-designated Solo or via the dual-medication port adaptor of the i-*AIRE* (Fig. 3). The continuous infusion function was quantified by fitting data to a straight line by simple linear regression for each period where radioactivity was due only to continuous infusion, with each infusion slope containing ∼7–15 data points (Table 3 and Fig. 5).

**FIG. 4.**
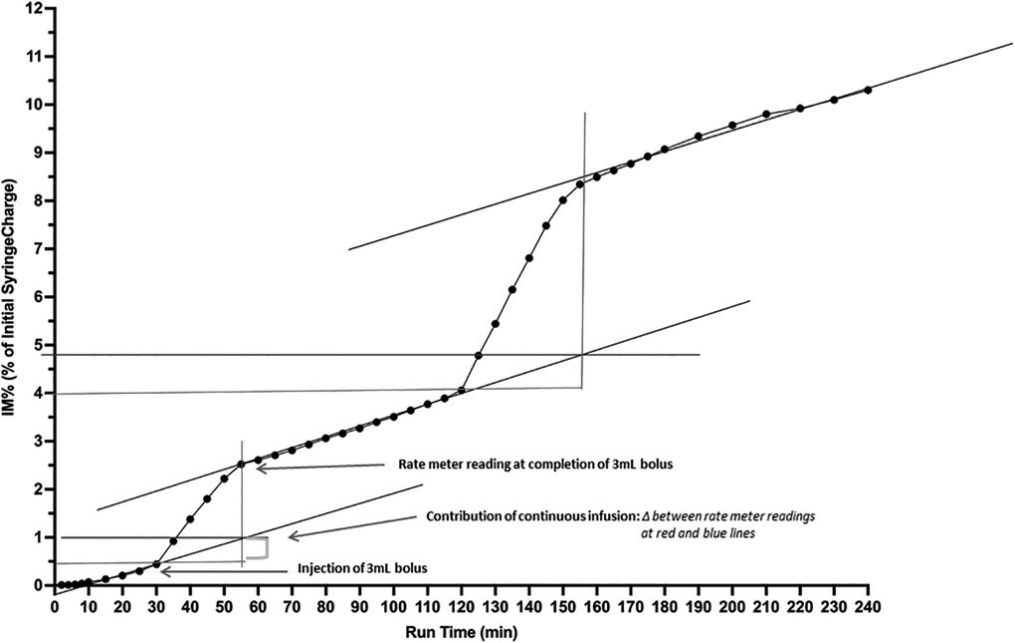
Depicts *i-AIRE* BEJN at 5 mL/h and DC 0.13, including slopes and quantification of contribution of continuous infusion used for calculation of IM% from bolus syringe deposited on filter. Vertical axis: IM%, expressed as a % of initial syringe charge; Horizontal Axis: run time (minutes). DC, duty cycle; IM, inhaled mass.

For the bolus experiments, the changes in radioactivity were quantified by measuring the step up in activity following bolus injection and subtracting the extrapolated contribution to aerosol production for the continuous infusion with each bolus experiment containing between 3 and 15 data points depending on length of bolus treatment (Fig. 4). The time of nebulization of the bolus was estimated by the period over which the slope of the activity line returned to that of only continuous infusion. With the continuous infusion baseline re-established, the second bolus (6 mL) was injected at 120 minutes and a similar calculation performed to quantify aerosol delivery from the 6 mL bolus to the IM filter.

This protocol was followed for each ventilator setting and pump flow. Eight 4-hour infusions were performed each with a 3 and 6 mL injected bolus. Data from this study were compared with results from a previous publication from our group^7^ where bolus delivery was tested without being superimposed on continuous infusion. Bolus data were compared using Mann Whitney, linear regression was used to derive continuous infusion slopes, and Kruskal Wallis for analysis of continuous infusion slopes (GraphPad Prism 9.0 for Mac OS; GraphPad Software, San Diego, CA). A *p*-value of <0.05 was considered statistically significant for all tests.

**FIG. 5.**
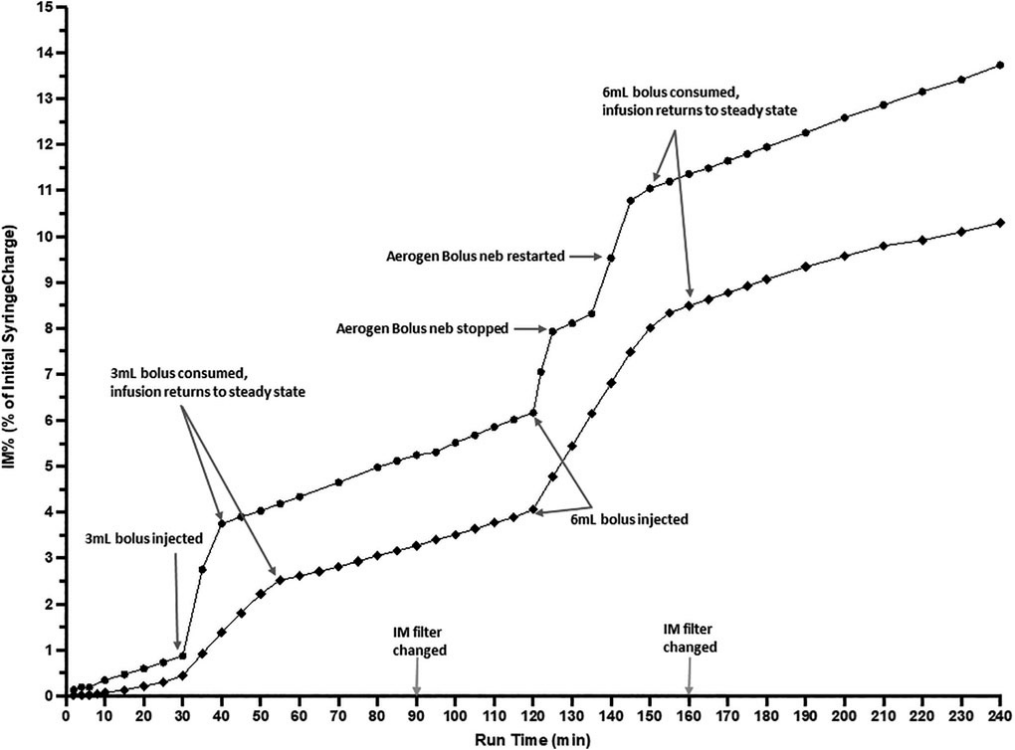
i-*AIRE* BEJN (■) and Aerogen Solo VMN (•) during continuous infusion at 5 mL/h and DC 0.13. Three milliliters bolus injected into nebulizer at 30 minutes, 6 mL bolus added at 120 minutes. IM filter changed at 90 and 160 minutes. Aerogen Solo VMN: 6 mL bolus nebulizer spontaneously stopped at 125 minutes, tapped and restarted at 135 minutes with bolus consumed by 145 minutes. IM filter changed at 90 and 160 minutes. Vertical Axis: IM%, expressed as a % of initial syringe charge; horizontal axis: run time (minutes).

For graphs (Figs. 4 and 5), the vertical axis represents captured radioactivity on the IM filter plotted as a percent of the initial syringe charge. The horizontal axis is time of nebulization in minutes. Each data point was calculated by converting the CPM measured by the ratemeter into μCi. Ratemeter counts were converted into radioactivity at the end of the experiment by measuring final activity on the IM filter. Details for this calculation are reported in the study by McPeck et al.^9^

### Calculation of bolus IM%

Black lines were drawn through three slopes indicating periods of continuous infusion. Three and 6 mL bolus injections were performed at 30 minutes (3 mL) and 120 minutes (6 mL) as indicated by the horizontal red line passing through the point at 30 and 120 minutes, respectively. After a bolus was introduced, the graph shifted markedly during the bolus nebulization and the red line extended horizontally for the duration of the bolus delivery. The red perpendicular line extends to the data point where the bolus was consumed and the continuous infusion slope consistent with the original steady state was re-established. The blue horizontal line is drawn at the point where the infusion slope and the bolus introduction intersect providing quantification of the contribution of the continuous infusion to the bolus.

## Results

### IM during bolus delivery

Data for bolus delivery (Table 2) were expressed as IM (percent of BOLUS syringe charge) deposited on the filter. Bolus delivery for i-*AIRE* BEJN and Aerogen VMN mean ± standard deviation (SD) ranged from 13.1% ± 0.3% to 34.9% ± 1.9% and 19.3% ± 2.8% to 44.9% ± 1.8%, respectively. Kruskal Wallis analysis comparing bolus data from current study to that of Lee et al.^7^: (3 mL/DC 0.13: BEJN *p* = 0.267, VMN *p* = 0.071; 6 mL/DC 0.13: BEJN *p* = 0.133, VMN *p* = 0.056; 3 mL/DC 0.34: BEJN *p* = 0.095, VMN *p* = 0.089; 6 mL/DC 0.34: BEJN *p* = 0.133, VMN *p* = 0.056) (Table 2).

**Table 2.** Analysis of Inhaled Mass Percent for Bolus Delivery for Current Study and Lee and Colleagues^9^

Nebulizer	Current study (simultaneous delivery)			Lee and colleagues^9^ (isolated bolus delivery)			Mann Whitney analysis of IM% for current study and Lee and colleagues^9^		
	Bolus vol. (mL)	IM% ± SD		Bolus vol. (mL)	IM% ± SD		Bolus vol. (mL)	*p*	
		DC 0.13	DC 0.34		DC 0.13	DC 0.34		DC 0.13	DC 0.34
i*-AIRE*	3	13.1 ± 0.3	34.9 ± 1.9	3	13.7 ± 2.5	14.5 ± 1.7	3	0.267	0.095
	6	18.1 ± 0.9	31.0 ± 1.2	6	15.5 ± 4.0	19.9 ± 4.5	6	0.133	0.133
Aerogen	3	19.3 ± 2.8	34.9 ± 1.9	3	13.6 ± 2.0	25.5 ± 5.7	3	0.071	0.089
	6	22.4 ± 3.4	44.9 ± 1.8	6	13.4 ± 3.4	19.3 ± 5.9	6	0.056	0.056
	DC 0.13: VC-CMV rate 15, VT 450 mL, Ti 0.5 seconds, PEEP +5 DC 0.34: VC-CMV rate 20, VT 650 mL, Ti 1.0 seconds, PEEP +5								

Analysis of IM% for bolus delivery with Mann Whitney analysis comparing bolus data from current study to that of Lee and colleagues.

IM%, inhaled mass percent; SD, standard deviation.

### IM during continuous infusion

IM during continuous infusion increases linearly as a function of time for both devices (mean ± SD: 2.1% ± 1.5%, 2.5% ± 0.9% for BEJN and VMN, respectively) (Figs. 4 and 5). Linear regression derived the three infusion slopes during continuous infusion for each experimental condition before and after each bolus injection (Table 3) with slopes ranging from 0.009 to 0.090. Kruskal Wallis analysis (Table 3) comparing the slopes for both nebulizers under the same experimental conditions: (10 mL/h, DC 0.34: *p* = 0.533; 10 mL/h, DC 0.13: *p* = 0.933; 5 mL/h, DC 0.34: *p* = 0.667; 5 mL/h, DC 0.13: *p* = 0.667). Comparison of all slopes for both nebulizer technologies for all experimental conditions (DC, pump flow): *p* = 0.983 (Table 3).

**Table 3.** Slopes of Continuous Infusion with Kruskal Wallis Analysis

Aerogen VMN	Run 1: 10 mL/h, DC 0.34			Run 2: 10 mL/h, DC 0.13			Run 3: 5 mL/h, DC 0.34			Run 4: 5 mL/h, DC 0.13		
	Slope 1	Slope 2	Slope 3	Slope 1	Slope 2	Slope 3	Slope 1	Slope 2	Slope 3	Slope 1	Slope 2	Slope 3
Slope	0.090	0.090	0.090	0.083	0.059	0.057	0.030	0.038	0.038	0.026	0.030	0.030
*n*	8	13	13	8	15	13	8	13	15	7	13	13

i-AIRE BEJN	Run 5: 10 mL/h, DC 0.34			Run 6: 10 mL/h, DC 0.13			Run 7: 5 mL/h, DC 0.34			Run 8: 5 mL/h, DC 0.13		
	Slope 1	Slope 2	Slope 3	Slope 1	Slope 2	Slope 3	Slope 1	Slope 2	Slope 3	Slope 1	Slope 2	Slope 3
Slope	0.070	0.061	0.060	0.034	0.046	0.065	0.009	0.011	0.013	0.013	0.023	0.023
*n*	7	13	13	8	15	13	8	15	13	7	13	13

Kruskal Wallis analysis	*p* = 0.533,* n* = 6			*p* = 0.933,* n* = 6			*p* = 0.667,* n* = 6			*p* = 0.667,* n* = 6		
Condition	10 mL/h, DC 0.34			10 mL/h, DC 0.13			5 mL/h, DC 0.34			5 mL/h, DC 0.13		
Condition	All slopes, all pump flows and DCs: *p* = 0*.983*, *n* = 24											

Linear regression derived the three infusion slopes during continuous infusion for each experimental condition before and after each bolus injection. Kruskal Wallis analysis compared the slopes for both nebulizers under the same experimental conditions as well as all nebulizer technologies, all slopes for all experimental conditions.

i-*AIRE* BEJN protocols were routinely completed without incident with a typical experiment shown in Figures 4 and 5. Several Aerogen VMN experiments were intermittently interrupted by random interruptions in nebulization with continuous infusion VMN delivery stopping spontaneously in 3 of 8 runs (38%) and bolus VMN delivery stopping spontaneously in 3of 16 runs (19%), an example is illustrated in Figure 5. Tapping restarted VMN function during continuous and bolus delivery runs (Fig. 5), and Figure 5 demonstrates sample tracings of combined aerosol therapy for the i-*AIRE* BEJN and Aerogen Solo VMN as well as the responsiveness of the system, when the VMN spontaneously stopped, was tapped and restarted.

### Limitations

This was an *in vitro* bench study and does not assess patient-related factors or define actual clinical aerosol delivery using these systems. Our data set the stage for clinical trials and support the case report of Elnadoury et al.^1^ who first modified a ventilator circuit using stacked Aerogen Solo VMN devices. Radioactive saline was measured in this study, not actual drug delivery. However; normal saline has been shown to be a suitable surrogate for multiple drugs.^10–14^ The observation period of 240 minutes may not describe long-term continuous nebulization on the order of days. Only two breathing patterns, chosen as the extreme of clinically relevant settings, and two pump flows were studied. This article serves as a detailed template for future studies

## Discussion

This study demonstrates that it is possible to deliver two aerosols simultaneously to an intubated patient without interrupting the ventilator circuit. Aerosol delivery for continuous infusion as well as the individual bolus was quantified separately supporting the concept that the clinician can interpret a dose-response to a given treatment. Previous reports have provided detailed comparisons between the nebulizer technologies.^7,8,16^ This study was designed to test the behavior of simultaneous bolus injections superimposed on continuous drug delivery. Two different nebulizer configurations were tested. Data for the Aerogen Solo VMN quantified aerosol delivery using the method first proposed in the case report of Elnadoury et al.^1^ who illustrated the technique of stacking two Aerogen Solo VMN devices in a single circuit. These data were compared with a different technology, the InspiRx i-*AIRE* BEJN which received both the continuous infusion and bolus injection into the same chamber.

The real-time ratemeter tracings indicated that behavior was similar for both technologies. Devices were monitored quantitatively by the ratemeter and independently by visual inspection to ensure that they completely emptied during bolus delivery and did not fill during continuous infusion. If the VMN spontaneously stopped, it was tapped to restart facilitating complete emptying. The latter maneuver increased overall delivery resulting in small but significant differences in bolus delivery (Table 2) between the two technologies. Previous studies by our group^7,8^ did not tap to restart the VMN and did not detect overall differences in function. The VMN tends to deliver a higher average dose when it completely empties.^7,8^

Data from the present study indicate that the slopes during continuous infusion are not different between the two technologies consistent with the concept that, unlike during a bolus, the continuous infusion is a “drop by drop” method and delivery is not affected by nebulizer output or residual.^7,16^ Once the nebulizer reaches a steady state, what is dripped into the nebulizer is completely nebulized provided that the drop-by-drop rate (pump flow) does not exceed the nebulizer's output.^7,8,16^ For continuous therapy, the Aerogen VMN devices produced similar ratemeter traces to the i-*AIRE* BEJN (Fig. 5), consistent with the drop-by-drop method. Examination of the initial slope tracings reveals that the Aerogen VMN tends to have a faster start to nebulization in the first 5-to-10 minutes of treatment with both technologies achieving similar slopes thereafter (Table 3).

With the introduction of the bolus, aerosol was delivered at the maximal output of either the single i*-AIRE* BEJN or the stacked Aerogen Solo VMN that received the bolus. The qualitative behavior of the single i-*AIRE* BEJN was similar to the two Aerogen Solo VMN, indicating that the transients for the bolus injections were similar such that the devices could be used interchangeably in a clinical situation provided that the two solutions are compatible. Particle size for both nebulizers has been described in detail in previous articles by our group: McPeck et al. measured ∼2.0 μm during continuous infusion while Ashraf et al. measured ∼2.0 μm for bolus delivery during humidification.^7–9^

The present study demonstrates that both radioactive saline solutions (bolus and continuous infusion) are delivered in a predictable manner. That is, the infusion was delivered at its prescribed rate and the bolus at the maximal output rate of the nebulizer. In a clinical situation, the infusion works in the background as the bolus drug is delivered. For both nebulizer types, all the drugs that are placed in the nebulizer are consumed and the nebulizer should not be filled during therapy. After the bolus was consumed the infusion slope returned to its prior continuous rate. This observation was confirmed by visual inspection. The real-time ratemeter technique allowed visualization of transients during nebulization, including interruptions in delivery due to spontaneous cessation of nebulization, as well as quantification of both the bolus and infused radioactive surrogate drugs.

In the analysis of the dynamic changes in the output graphs seen during introduction of the bolus during stacked (VMN) or single (BEJN), results were similar regardless of delivery method and consistent with the observations of isolated bolus delivery by Lee et al.^7^ Mixing of drugs within a nebulizer could result in solution incompatibilities, but it is known that common drugs such as bronchodilators and steroids can be safely mixed.^15^ Our data support the use of the i-*AIRE* BEJN with dual-medication port interchangeably with two stacked Aerogen Solo VMN. Either of the two nebulizer technologies can be used to deliver multiple aerosols simultaneously and that concurrent bolus delivery does not change the overall qualitative and quantitative behavior of the continuous infusion.

A clinician wishing to treat a patient with two drugs simultaneously might ask the following questions: When you inject the bolus, what happens? Does introduction of the bolus interrupt the infusion? Is bolus delivery using the current method different than conventional stand-alone bolus delivery? The current study demonstrates that treatment using the two drugs occurs independently and in the classic manner, the infusion runs continuously in the background at its prescribed rate evidenced by the slopes and the bolus would be delivered immediately as the nebulizer functions at maximum output. In conclusion, simultaneous multidrug delivery is possible and opens a broader spectrum of possibilities for aerosol delivery in the clinical setting.

## Notes

Reviewed by:

Laurent Vecellio

Ariel Berlinski
